# Effects of seasonal grazing on plant and soil microbial diversity of typical temperate grassland

**DOI:** 10.3389/fpls.2022.1040377

**Published:** 2022-11-03

**Authors:** Chun-Hui Ma, Xing-Hai Hao, Feng-Cai He, Tao-Getao Baoyin, Jue-Jie Yang, Shi-Kui Dong

**Affiliations:** ^1^ School of Grassland Science, Beijing Forestry University, Beijing, China; ^2^ School of Ecology and Environment, Inner Mongolia University, Hohhot, China

**Keywords:** grassland, seasonal grazing, plant composition, plant diversity, soil microbial diversity

## Abstract

Biodiversity is the decisive factor of grassland ecological function and process. As the most important human use of grassland, grazing inevitably affects the grassland biodiversity. However, comprehensive studies of seasonal grazing on plant and soil bacterial, archaeal and fungal diversity of typical temperate grassland are still lacking. We examined the impact of seasonal grazing, including no-grazing (NG), continuous grazing (CG), grazing in May and July (G57), grazing in June and August (G68), and grazing in July and September (G79) on grassland plant and soil microbial diversity based on a long-term field grazing experiment. The results showed that the aboveground plant biomass (AGB) of the seasonal grazing plots was significantly higher than that of the CG plots. Compared with NG, CG increased significantly the Margalef richness index of plant community, while did not significantly change the Shannon, Simpson and Pielou evenness of plant community. Grazing changed the composition and biomass of dominant vegetation. Long-term grazing decreased the proportion of *Leymus chinensis (Trin.) Tzvel.* and increased the proportion of *Cleistogenes squarrosa (Trin.) Keng.* There was no significant change in the Shannoneven, Shannon and Coverage indices of soil bacteria, archaea and fungi between NG and the grazing plots. But the Chao index of soil fungi in G57, G68 and G79 and archaea in G57, G79 was significantly higher than that in CG. The results of correlation analysis showed that the plant diversity in the CG plots was significantly negatively correlated with the soil bacterial diversity. The plant richness in the G57 and G68 plots was significantly positively correlated with the soil archaea richness. Our study showed that seasonal grazing was a sustainable grazing management strategy for maintaining typical grassland plant and soil microbial diversity in northern of China.

## Introduction

Grassland is one of the most species rich ecosystems on earth, and its species diversity is no less than that of tropical rainforest (Pulungan et al., 2019). Grazing is the most important human factor affecting grassland biodiversity and ecosystem functions (Hou and Yang, 2006). The selective feeding of herbivores on plants and different feeding frequencies and intensities affected grassland plant diversity and productivity (Zhang et al., 2018). Previous studies about the impact of grazing on grassland biodiversity are inconsistent. Grazing increased the spatial heterogeneity of resource distribution to improve species diversity (Hou and Yang, 2006), and promoted species equity by eliminating competitive advantages (Sternberg et al., 2015). The results of Suo et al. (2022) on the alpine grassland in Northwest China showed that overgrazing and enclosure will reduce the diversity and productivity of plant communities. Duan et al. (2010) studied the typical temperate grassland in Inner Mongolia and showed that moderate grazing would increase grassland species diversity, while overgrazing would reduce grassland community diversity and productivity. These studies mainly focused on the impact of grazing intensity on grassland ecosystem diversity, and paid less attention to the impact of grazing system (such as continuous grazing, seasonal grazing, rotational grazing, etc.) on grassland biodiversity.

Since 2000, a series of grassland protection programs based on grazing prohibition, seasonal grazing and rotational grazing strategy, including “Grain for Green Program”, “Grassland Ecological Protection Program”, and “Beijing-Tianjin Sandstorm Source Control Project”, were launched to protect and make sustainable use of grasslands in China (Cai et al., 2020). Seasonal grazing (rest grazing) could balance the forage production and livestock growth, and is an important grazing strategy to promote grassland ecological protection and rational utilization of grassland resources. Liu et al. (2016) found that the plant diversity of warm season grazing grassland was higher than that of cold season grazing grassland. Due to phenology of plants, grazing in summer and autumn tended to reduce the evenness of grassland, while grazing in spring was conducive to the growth of dominant species and the biomass accumulation of grassland community on the typical temperate grassland (Zheng, 2020). However, there were few studies on the response and mechanism of aboveground and underground biodiversity under seasonal grazing.

Soil biodiversity is an important component of grassland biodiversity. Grazing would change the composition and diversity of grassland soil organisms, especially microorganisms (Hu et al., 2015; Van Syoc et al., 2022). On the one hand, the trampling of livestock and fecal input could change the soil physical structure and chemical composition, thereby affecting the microbial community structure (Clegg, 2006; Qu et al., 2016). On the other hand, livestock changed the composition of plant community, underground rhizome distribution and root exudates through selective feeding, and affected the composition of soil microbial community (Fichtner et al., 2014; Steinauer et al., 2016; Zhang et al., 2019). He et al. (2009) found that the diversity of soil microbial communities in 5 vegetation types showed significant differences in species richness index and uniformity index, while Shannon index did not differ significantly. This suggests that the composition of aboveground plant communities affects the α diversity of soil microorganisms. Recent study showed that under heavy grazing, the soil carbon and nitrogen nutrient cycling significantly reduced, resulting in the change of beta-diversity of soil bacteria and fungi (Zhang and Fu, 2021). Light grazing can effectively increase soil microbial α diversity, while heavy grazing reduces soil microbial alpha-diversity (Xun et al., 2018). At the same time, heavy grazing can alter the proportion of bacteria and fungi in the soil, with an increase in bacteria and a decrease in fungi (Xun et al., 2018). This may be due to the main groups of fungi are known as arbuscular mycorrhizal fungi (AMF), is closely linked to plant biomass, so heavy grazing is not conducive to the colonization of these fungi (Yang et al., 2018), while bacteria proliferate rapidly (Klumpp et al., 2009; Nelson and Carlson, 2012). Yang et al. (2022) argue through meta-analysis that heavy grazing continuously reduces microbial biomass and abundance, and that the reason for this result is that grazing reduces plant aboveground biomass and increases soil volume. But Van Syoc et al. (2022) found that there was no significant difference in soil microbial diversity between grazing and non-grazing plots. Some other studies have shown that two years grazing increased the richness of soil bacteria (Epelde et al., 2017; Song et al., 2020). However, there are lacking studies about the impact of long-term seasonal grazing on grassland soil microbial diversity.

We hypothesized that there might be significant differences in the composition and diversity of plant and soil microbial communities under different grazing systems (grazing strategies), and the diversity of soil microorganism and plant community could be consistent with long-term grazing. To verify these hypotheses, we analyzed the impact of seasonal grazing (five grassland management treatments) on plant biomass, plant community composition and diversity and soil microorganisms (including bacteria, archaea and fungi) based on long-term field grazing experiments in a typical temperate grassland. We further studied the relationship between aboveground and underground biodiversity under different grazing systems (grazing strategies).

## Materials and methods

### Study site

The field study was implemented at the Grassland Ecosystem Research Station of Inner Mongolia University in Xilinhot City, Xilingol League, Inner Mongolia Autonomous Region (44°9′18′′N, 116°23′32′′E). This region was characterized by a temperate semi-arid continental climate, with annual mean temperature and precipitation of -0.4°C and 271 mm respectively (Chen et al., 2021). The region’s typical grassland covers approximately 85% of the area, and the aboveground vegetation was dominated by *Stipa krylovii Roshev*, *Leymus chinensis (Trin.) Tzvel.*, *Cleistogenes squarrosa (Trin.) Keng*. The soil type is chestnut soil with a sandy loam texture. The growing season usually starts in early May and ends in late September in this region (Song et al., 2020).

### Experimental design

The grazing experiments with five grassland management treatments was launched in 2012: no-grazing (NG), continuous grazing (from May to September, CG), grazing in May and July (grazing started in the spring, G57), grazing in June and August (grazing started in the early summer, G68), and grazing in July and September (grazing started in the late summer, G79). Three repetitions were randomly set for each treatment, and the area of each plot was 33.3 m × 33.3 m. Six sheep were released in the grazing plot on the 21st day of each month and stopped when the mean plant height about 6 cm for protecting the grass from overgrazing damage (moderate grazing).

### Sampling and measurement

Plant and soil samples were collected at the end of September in 2021. Within each plot, plant communities were surveyed using three 1 × 1 m quadrats to measure the abundance, coverage and height of plants. Then standing plants of each species was clipped to 1 cm above the ground, and oven-drying at 80°C for 24 h to calculate plant biomass. Total plant aboveground biomass (AGB) was calculated as the sum of all species biomass.

The soil samples (0-20 cm) for DNA analysis were collected in each quadrat and transported on dry ice to the laboratory, and stored at –80°C before analysis. The soil DNA was extracted using PowerSoil DNA extraction kit (MoBio Laboratories, CA) according to the manufacturer’s instructions. For 16S rRNA sequencing, the universal primers of bacteria (338F and 806R, ACTCCTACGGGAGGCAGCAG; GGACTACHVGGGTWTCTAAT), archaea (Arch524F and Arch 958R, TGYCAGCCGCCGCGGTAA; YCCGGCGTTGAVTCCAATT), and fungus (ITS1F_ITS2R, CTTGGTCATTTAGAGGAAGTAA;GCTGCGTTCTTCATCGATGC) were used for PCR. PCR products were pooled, purified, sequenced on an Illumina MiSeq platform by Majorbio Corporation, Shanghai, China. Sequence data were analyzed using QIIME 2 and to calculate alpha-diversity indices.

The formulas of alpha-diversity indices were shown in Supporting Information Method S1, including Margalef, Shannon, Simpson, Pielou evenness, Chao, Shannoneven and coverage. The beta-diversity of soil microorganism was then visualized with R language (version 3.3.1).

### Statistical analysis

The results were analyzed using the SPSS software with version 23.0 (SPSS Inc., Chicago, IL, USA). Differences in characteristics of plant communities (the biomass, abundance, coverage, height and diversity) and soil microbial diversity among five grassland management treatments were analyzed using one-way analysis of variance (ANOVA). The alpha-diversity indices of plant were characterized by Margalef, Shannon, Simpson and Pielou evenness index (Thukral, 2017). Differences were considered statistically significant when P < 0.05. The alpha-diversity and beta-diversity (Principal coordinates analysis, PCoA) of plant and soil microorganism was analyzed and visualized by the “Vegan” package within R (http://cran.r-project.org/web/packages/vegan).

## Results

### Plant composition, productivity, height and diversity

**Figure 1:**
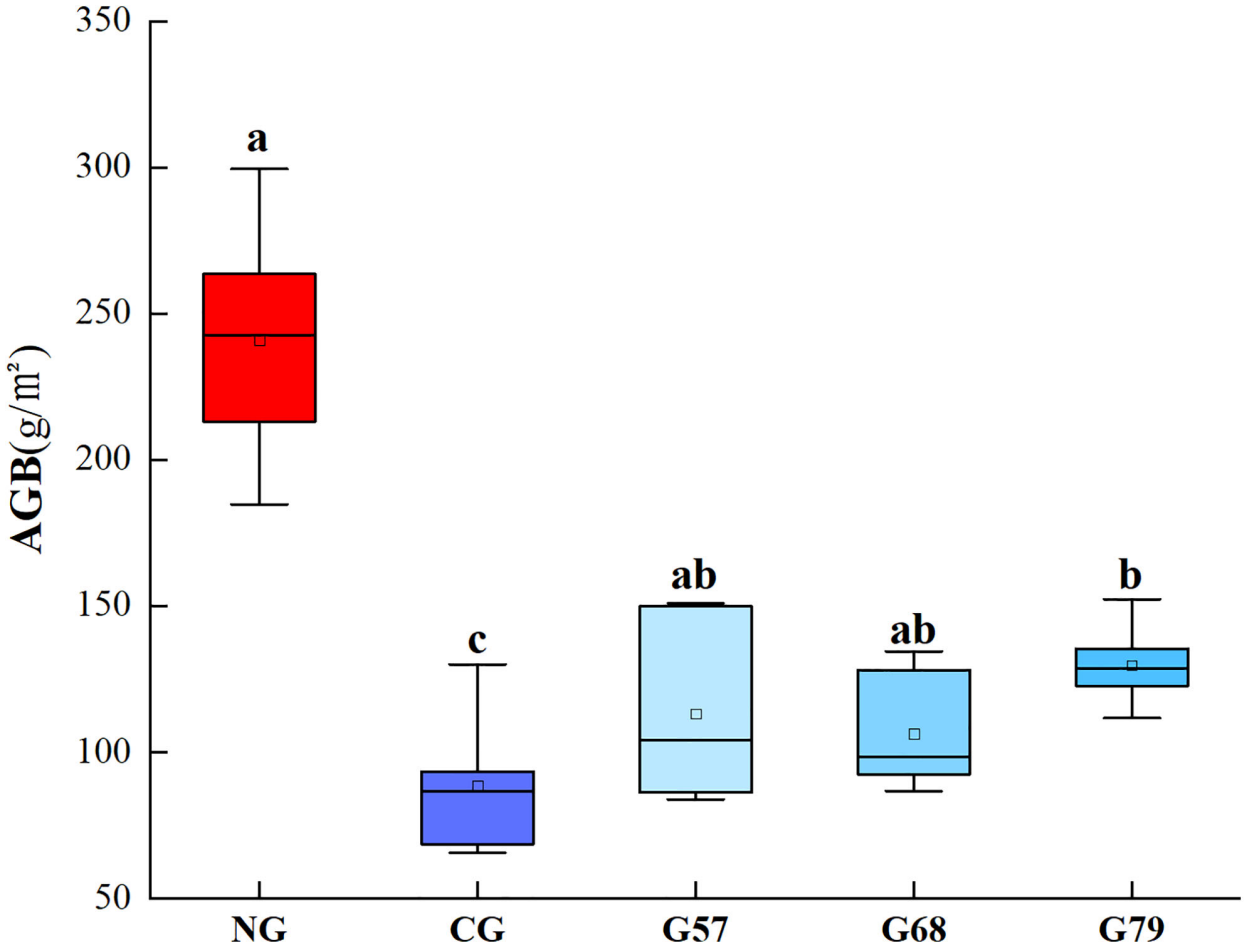
Aboveground plant biomass (AGB) in five grassland management treatments. NG: No grazing; CG: Continuous grazing; G57: Grazing in May and July; G68: Grazing in June and August; G79: Grazing in July and September. The error bars in the figure represent the standard error, and the lowercase letters represent the significant differences (*P*<0.05).

The plant biomass of grassland in five grassland management treatments was shown in **Figure 1**. The AGB of no-grazing (NG) was significantly (*P* < 0.05) higher than that of continuous grazing (CG) and seasonal grazing (G57, G68, G79). The aboveground plant biomass of seasonal grazing was significantly higher than that of CG (*P* < 0.05). There was no significant difference in the AGB among G57, G68 and G79 (*P* > 0.05).

**Figure 2:**
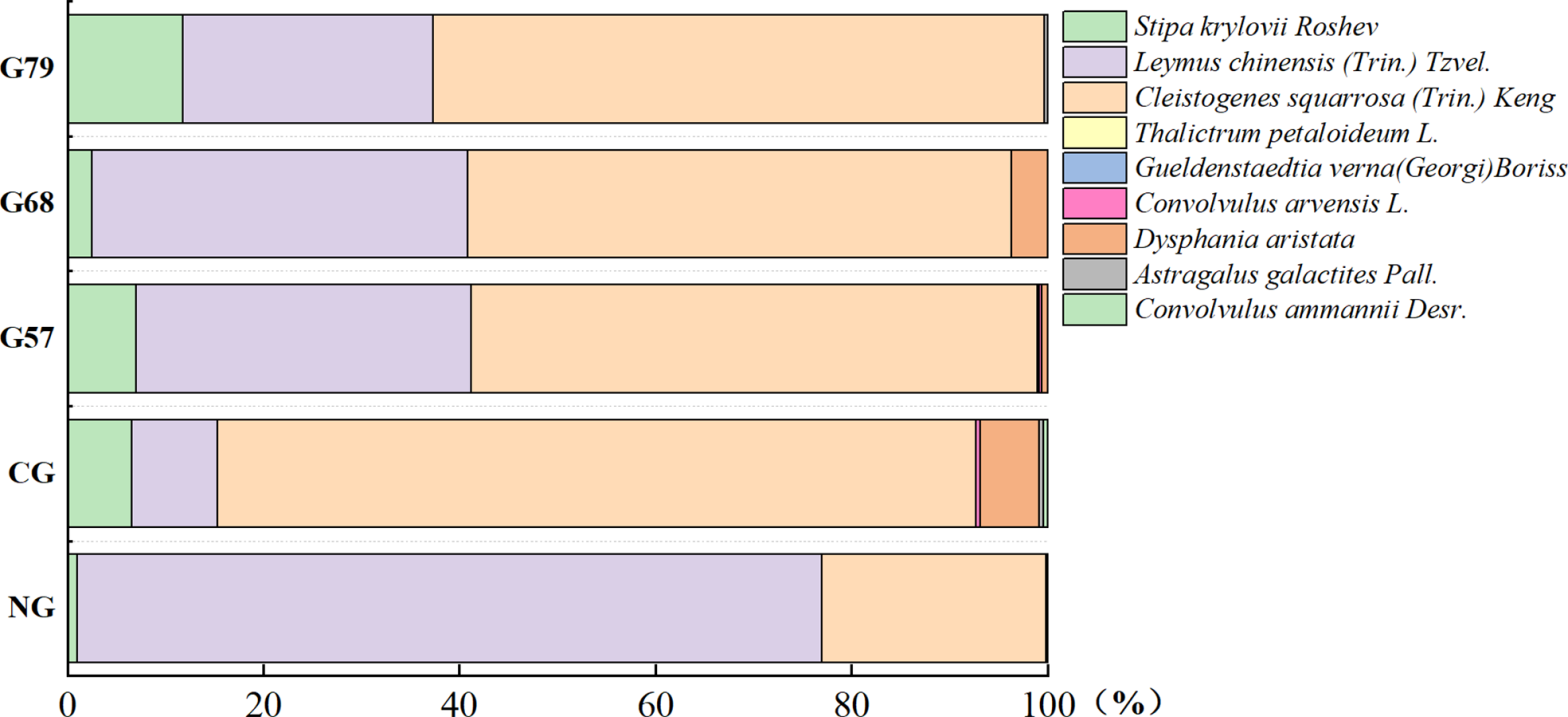
Percentage stacked columnar graph of aboveground plant species in five grassland management treatments. NG, CG, G57, G68, G79. NG: No grazing; CG: Continuous grazing; G57: Grazing in May and July; G68: Grazing in June and August; G79: Grazing in July and September.

**Figure 3:**
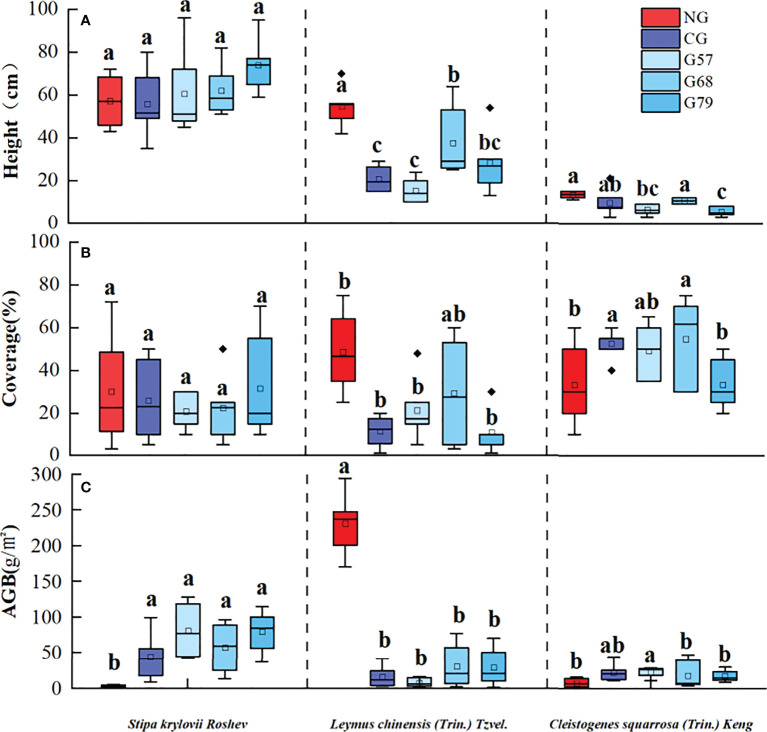
Grassland community characteristics in five grassland management treatments: **(A)** height, **(B)** coverage, **(C)** AGB. NG: No grazing; CG: Continuous grazing; G57: Grazing in May and July; G68: Grazing in June and August; G79: Grazing in July and September. The error bars in the figure represent the standard error, and the lowercase letters represent the significant differences (*P*<0.05).

After 10 years of moderate grazing, the dominant species of the aboveground plant community have not changed, namely *Stipa krylovii Roshev*, *Leymus chinensis (Trin.) Tzvel.* and *Cleistogenes squarrosa (Trin.) Keng* remained as the dominant species (**Figure 2**). However, the proportion of dominant species has changed significantly with grazing systems(grazing strategy). Compared with the no-grazing, the proportion of *L. chinensis* in the grazing plots decreased. In CG, *L. chinensis* decreased and *C. squarrosa* increased greatly. There was no significant difference in the height and coverage of *S. krylovii* in grazing and no-grazing plots (*P* > 0.05), but the biomass of *S. krylovii* in grazing plots was significantly higher than that in no-grazing plots (*P* < 0.05, **Figure 3**). The height and biomass of *L. chinensis* in grazing plots were significantly lower than that in no-grazing plots (*P* < 0.05). The coverage of *C. squarrosa* in G68 was significantly higher than that in NG (*P* < 0.05).

**Figure 4:**
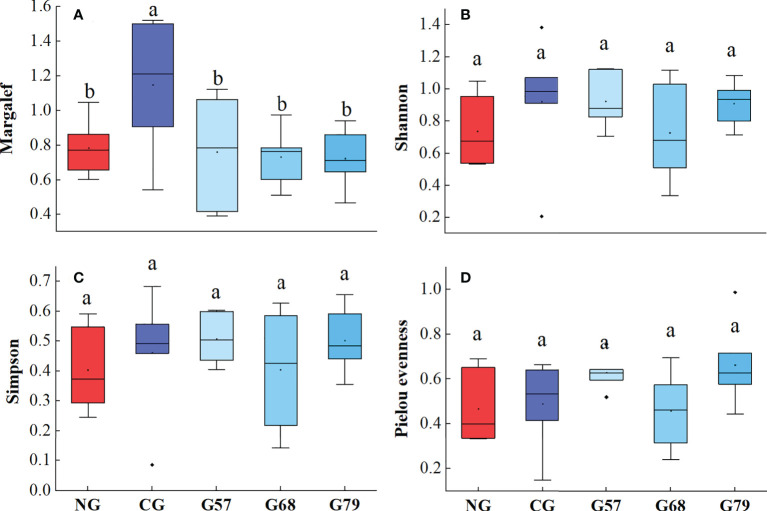
Aboveground plant diversity indicators in five grassland management treatments: **(A)** Margalef; **(B)** Shannon; **(C)** Simpson; **(D)** Pielou evenness. NG: No grazing; CG: Continuous grazing; G57: Grazing in May and July; G68: Grazing in June and August; G79: Grazing in July and September. The error bars in the figure represent the standard error, and the lowercase letters represent the significant differences (*P*<0.05).

The biodiversity indexes (Shannon, Simpson and Pielou evenness) of plants in different rest-grazing periods had no significant changes (*P* > 0.05), but the Margale richness index under CG was significantly (*P* < 0.05) higher than that under seasonal grazing (G57, G68, G79) and NG (**Figure 4**).

### Microbial diversity of grassland soil

**Figure 5:**
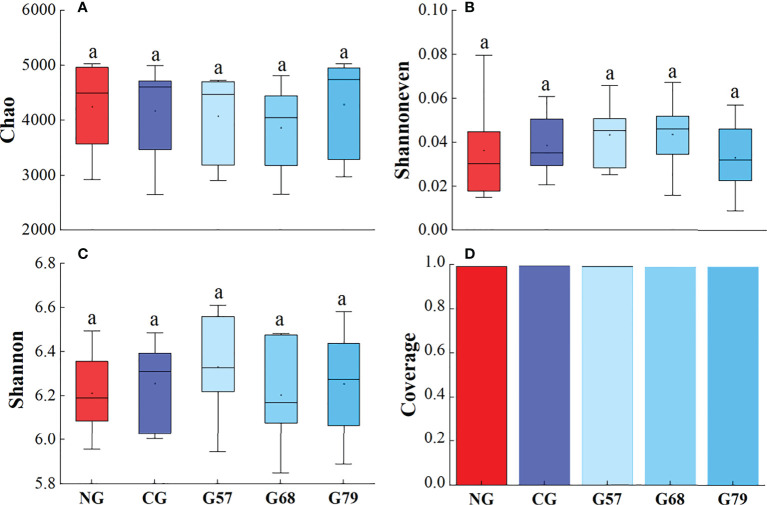
Soil bacterial diversity in five grassland management treatments: **(A)** Chao; **(B)** Shannoneven; **(C)** Shannon; **(D)** Coverage. NG: No grazing; CG: Continuous grazing; G57: Grazing in May and July; G68: Grazing in June and August; G79: Grazing in July and September. The error bars in the figure represent the standard error, and the lowercase letters represent the significant differences (*P*<0.05).

**Figure 6:**
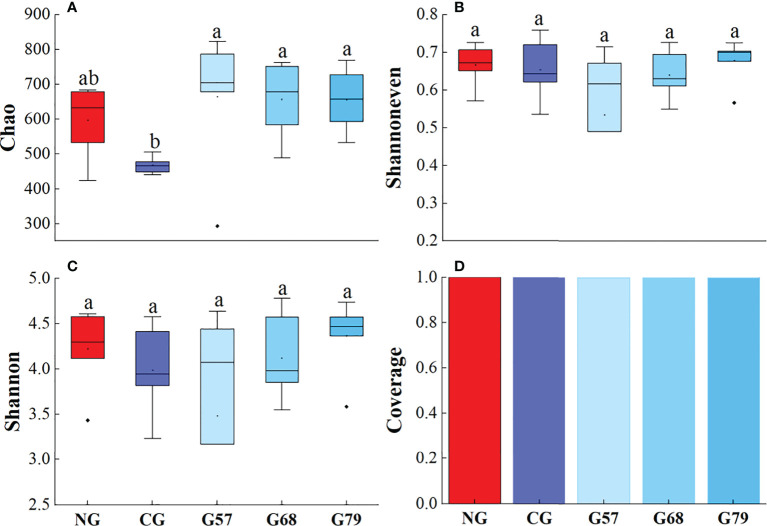
Soil fungal diversity in five grassland management treatments: **(A)** Chao; **(B)** Shannoneven; **(C)** Shannon; **(D)** Coverage. NG: No grazing; CG: Continuous grazing; G57: Grazing in May and July; G68: Grazing in June and August; G79: Grazing in July and September. The error bars in the figure represent the standard error, and the lowercase letters represent the significant differences (*P*<0.05).

**Figure 7:**
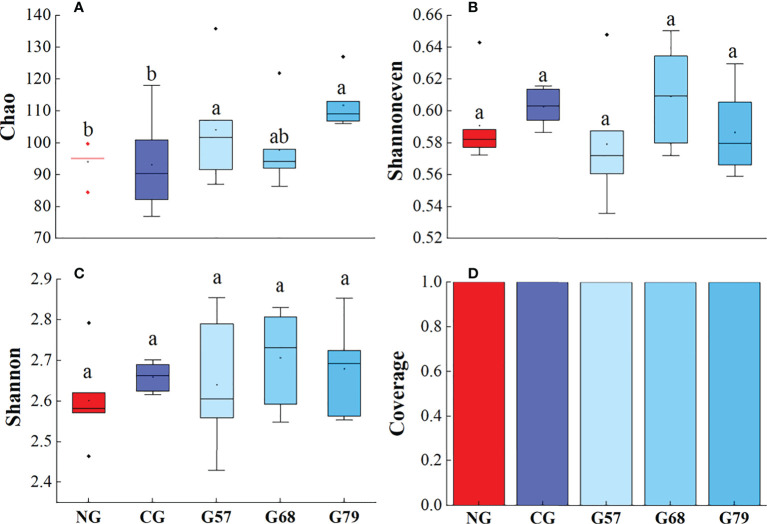
Soil archaeal diversity in five grassland management treatments: **(A)** Chao; **(B)** Shannoneven; **(C)** Shannon; **(D)** Coverage. NG: No grazing; CG: Continuous grazing; G57: Grazing in May and July; G68: Grazing in June and August; G79: Grazing in July and September. The error bars in the figure represent the standard error, and the lowercase letters represent the significant differences (*P*<0.05).

The Chao, Shannoneven, Shannon and Coverage indexes of soil bacteria in grazing and no-grazing plots had no significant changes (*P* > 0.05**, Figure 5**). The Chao index of soil fungi in seasonal grazing plots (G57, G68, and G79) was significantly higher than those in CG and NG plots (*P* < 0.05, **Figure 6**). Similarly, the Chao index of soil archaeal abundance in seasonal grazing plots (G57 and G79) was significantly higher than those in CG and NG plots (*P* < 0.05, **Figure 7**).

**Figure 8:**
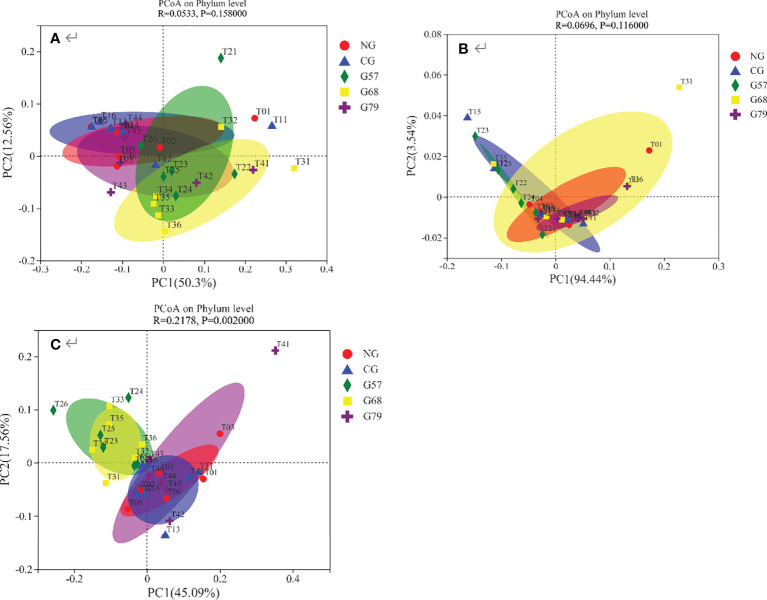
Soil microbial PCoA analysis in five grassland management treatments: **(A)** bacteria; **(B)** fungi; **(C)** archaea; NG: No grazing; CG: Continuous grazing; G57: Grazing in May and July; G68: Grazing in June and August; G79: Grazing in July and September. The error bars in the figure represent the standard error, and the lowercase letters represent the significant differences (*P*<0.05).

The results of PCoA showed that the contribution of the first principal coordinate axis of bacteria and fungi was 50.3% and 94.44%, respectively (**Figures 8** **A, B**). The composition of bacteria and fungi was all similar in five grassland management treatments. The contribution of the first principal coordinate axis of soil archaea was 45.09%, and the composition of archaeal community were separated between NG and G57 indicating beta-diversity was different between them (**Figure 8** **C**).

### Correlation analysis of plant and soil microbial diversity

The results of correlation analysis showed that there was no significant correlation between the aboveground plant diversity and the diversity of soil fungi, archaea and bacteria in NG plots (*P* > 0.05, **Table 1**). In CG plots, the Shannon and Pielou evenness indexes of aboveground plants were significantly negatively correlated with the Shannon and Shannoneven indexes of soil bacteria (*P* < 0.05). In G57 and G68 plots, the Chao index of soil archaea had significant positive correlations with the Simpson and Margalef index of plants, respectively (*P* < 0.05, **Table 1**).

**Table 1:** Correlation analysis between grazing aboveground plants and soil microbial diversity in five grassland management treatments.

NG	Margalef (plant)	Shannon (plant)	Simpson (plant)	Pielou evenness (plant)
Chao(fungi)	-0.77	-0.03	0.31	0.31
Shannoneven(fungi)	-0.03	0.43	0.43	0.43
Shannon(fungi)	-0.09	0.14	0.20	0.20
Chao(archaea)	-0.09	0.54	0.49	0.54
Shannoneven(archaea)	-0.14	0.26	0.26	0.31
Shannon(archaea)	-0.09	0.14	0.14	0.14
Chao(bacteria)	0.09	-0.14	-0.14	-0.14
Shannoneven(bacteria)	-0.60	0.03	0.14	0.37
Shannon(bacteria)	-0.31	-0.14	-0.14	0.14
**CG**				
Chao(fungi)	-0.09	-0.03	-0.31	-0.31
Shannoneven(fungi)	0.37	0.09	-0.09	-0.09
Shannon(fungi)	0.09	0.03	-0.14	-0.14
Chao(archaea)	-0.03	0.37	0.77	0.77
Shannoneven(archaea)	-0.37	-0.66	-0.60	-0.60
Shannon(archaea)	-0.26	0.14	0.66	0.66
Chao(bacteria)	-0.37	-0.43	-0.26	-0.26
Shannoneven(bacteria)	0.09	-0.43	-0.83*	-0.83*
Shannon(bacteria)	-0.09	-0.83*	-0.96**	-0.96**
**G57**				
Chao(fungi)	-0.49	-0.37	-0.03	0.26
Shannoneven(fungi)	0.26	0.43	0.66	0.26
Shannon(fungi)	0.26	0.43	0.66	0.26
Chao(archaea)	0.54	0.71	0.94**	-0.14
Shannoneven(archaea)	-0.26	-0.54	-0.66	-0.43
Shannon(archaea)	-0.31	-0.31	-0.09	-0.03
Chao(bacteria)	-0.60	-0.26	-0.49	0.71
Shannoneven(bacteria)	-0.31	-0.43	-0.54	0.09
Shannon(bacteria)	-0.54	-0.31	-0.66	0.60
**G68**				
Chao(fungi)	-0.03	-0.14	-0.14	-0.14
Shannoneven(fungi)	-0.14	-0.60	-0.60	-0.60
Shannon(fungi)	-0.31	-0.49	-0.49	-0.49
Chao(archaea)	0.94**	0.31	0.31	0.31
Shannoneven(archaea)	-0.71	0.09	0.09	0.09
Shannon(archaea)	-0.03	0.77	0.77	0.77
Chao(bacteria)	0.03	-0.26	-0.26	-0.26
Shannoneven(bacteria)	-0.09	-0.60	-0.60	-0.60
Shannon(bacteria)	-0.31	-0.49	-0.49	-0.49
**G79**				
Chao(fungi)	0.14	-0.49	-0.49	-0.54
Shannoneven(fungi)	-0.71	0.31	0.31	0.43
Shannon(fungi)	-0.60	0.20	0.20	0.26
Chao(archaea)	0.20	-0.43	-0.43	-0.31
Shannoneven(archaea)	0.49	0.03	0.03	-0.09
Shannon(archaea)	0.37	0.14	0.14	0.09
Chao(bacteria)	0.14	-0.66	-0.66	-0.49
Shannoneven(bacteria)	-0.49	-0.09	-0.09	0.20
Shannon(bacteria)	-0.26	-0.37	-0.37	-0.09

NG, No grazing; CG, Continuous grazing; G57, Grazing in May and July; G68, Grazing in June and August; G79, Grazing in July and September (**, P<0.01;*, P<0.05).

## Discussion

### Effects of seasonal grazing on AGB and plant diversity

We found in present study that the AGB of seasonal grazing was significantly (*P* < 0.05) higher than that of continuous grazing. The possible reason might be that moderate grazing increased the nutrient supply and the photosynthetic intensity of the residual leaves, and increased the photosynthetic rate of the new leaves (Li, 1999). Livestock grazing on non-photosynthetic rhizomes and flowers could lead to more distribution of photosynthetic substances to leaves, thus activating dormant meristem and increasing plant biomass (Owen and Wiegert, 1976; McNaughton, 1979; Owen and Wiegert, 1981; Belsky, 1986). Under moderate grazing, the plants will appear over compensatory growth, which is the “grazing optimization hypothesis” (Mysterud et al., 2011).

We also found that different grazing systems(grazing strategy) had a significant impact on the height, coverage and biomass of aboveground dominant plants in the grassland. In order to adapt to the environment, plants could change their appearance in different environmental conditions to capture resources (Wang et al., 2010; Deng et al., 2014). In this study, the height of *C. squarrosa* was lower in NG plots, and it was obviously inferior to the tall grasses in the competition of light resources. Livestock selectively grazing on forage enhanced the light competition ability of *C. squarrosa*, and the coverage of *C. squarrosa* in the seasonal grazing plot (G68) was significantly higher than that in NG. Consistently, Wang and Wang (2001) found that moderate grazing promoted the compensatory growth of the population of *C. squarrosa*, and the coverage of *C. squarrosa* was significantly increased in the long-term grazing plots.

Here, the Margalef richness index of aboveground plant community in the continuous grazing plots (CG) increased significantly, but the Shannon, Simpson and Pielou evenness indexes did not change significantly. Compared with NG, the aboveground plant community diversity in grazing plots (CG, G57, G68, and G79) was not significantly different. This was similar to the results of Gu (2013) in the temperate mountain grassland of Yili Kazak Autonomous Prefecture of Xinjiang Uygur Autonomous Region, showing that moderate grazing did not decrease grassland plant biodiversity. Li et al. (2019) also found that grazing did not change the diversity of plants, but promoted the leaf characteristics and functional diversity of alpine meadow.

### Effects of seasonal grazing on soil microbial diversity

Because soil microorganisms could sensitively reflect the changes of grassland ecosystem functions, they were often used as an important indicator of soil ecosystem (Wardle et al., 2004). Eldridge et al. (2017) found that heavy grazing would reduce soil carbon content, thus affecting the abundance and diversity of soil actinomycetes and increasing the abundance of bacteria. Our results in this study showed that moderate grazing had no significant effect on soil bacterial abundance and diversity of typical grassland. Consistently, Wang et al. (2020) found that moderate grazing did not reduce grassland microbial biodiversity, but would help to maintain the diversity of plants, bacteria and fungi in the typical grassland of Inner Mongolia.

The Chao index of soil fungal richness in continuous grazing plots was significantly lower than that in seasonal grazing plots, which might be due to the competitive exclusion effect in our study. The species that was more effective in using resources led to reduce or elimination of other species from habitat (Hibbing et al., 2010). Grazing increased ascomycetes (dominant fungi) and led to the decrease of the abundance and diversity of other fungi (Zechmeister-Boltenstern et al., 2015). At the same time, moderate grazing improved the proportion of mycorrhizal plant species and made the fungal richness higher than that of continuous grazing (Ba et al., 2012). In addition, our study showed that the Chao index of archaea in seasonal grazing (G57, G79) plots was significantly higher than that in NG plots, which might be associated with the increase of soil ammonia oxidation archaea by grazing (Egan et al., 2018). However, due to the potential inhibitory effect of the herbivore urine on the abundance of soil ammonia oxidizing archaea (Egan et al., 2018), the abundance archaea under the continuous grazing treatment decreased compared with seasonal grazing.

### The relationship between plant and soil microbial diversity under different grazing strategies

We found that there was a significant correlation between the diversity of plants and soil microorganisms in grazing plots. The selection of herbivores changed the community structure and composition of plants, and also affected the soil microbial community structure (Bardgett et al., 1998; Gao et al., 2004). Due to grazing, grassland plants allocated more assimilated carbon to the root system (Hou and Yang, 2006), which stimulated the growth and activity of heterotrophic microorganisms in the rhizosphere (Bardgett and Cook, 1999). Recent studies have shown that plant diversity affected soil microbial communities by changing the amount and diversity of soil rhizosphere exudates (Zhang et al., 2019). Moreover, grazing affected the abundance of soil microorganisms by changing the amount of organic matter returned to the soil (Milchunas and Lauenroth, 1993). Gao et al. (2004) found that the soil microbial richness was the highest in moderate grazing, and there were significantly correlations between the diversity of plants and soil bacteria in the continuous grazing plots.

## Conclusions

Compared with continuous grazing, the seasonal grazing significantly increased the aboveground plant biomass of the temperate typical grassland, and the dominant species composition of the plants did not change significantly in this study. *S. krylovii*, *L. chinensis* and *C. squarrosa* were the dominant species. Long term moderate grazing increased Margalef richness index of aboveground plants and seasonal grazing had no significant effect on soil bacterial diversity. However, compared with continuous grazing, seasonal grazing can improve the Chao index of soil fungi and archaea. The diversity (Shannon and Pielou evenness indexes) of aboveground plants in the continuous grazing sample plots were significantly correlated with the Shannon and Shannon even indexes of soil bacteria. These results indicated that seasonal grazing, was helpful to maintain livestock productivity and biodiversity in the temperate grassland of Inner Mongolia, and should be preferentially used in the biodiversity protection and sustainable grazing management practice of the typical grassland in the northern temperate zone.

## Data availability statement

The original contributions presented in the study are publicly available. This data can be found here: https://www.ncbi.nlm.nih.gov/, accession number PRJNA894342.

## Author contributions

S-KD, J-JY, and T-GB planned and designed this study. F-CH, X-HH, helped to conduct the experiments. S-KD and J-JY revised this manuscript. C-HM analyzed the data and wrote this paper. All authors contributed to the article and approved the submitted version.

## Funding

This research was supported by the Yinshanbeilu Grassland Eco-hydrology National Observation and Research Station, China Institute of Water Resources and Hydropower Research, Beijing 100038, China, Grant NO. YSS2022017, Fundamental Research Funds for the Central Universities (2021ZY82), the National Natural Science Foundation of China (U20A2007, 72050001, 32201398) and the National Key R&D Program of China (2021FED1124000). We thank Cong Wang from Research Center for Eco-Environmental Sciences, Chinese Academy of Sciences for his comments and suggestions to improve our paper.

## Conflict of interest

The authors declare that the research was conducted in the absence of any commercial or financial relationships that could be construed as a potential conflict of interest.

## Publisher’s note

All claims expressed in this article are solely those of the authors and do not necessarily represent those of their affiliated organizations, or those of the publisher, the editors and the reviewers. Any product that may be evaluated in this article, or claim that may be made by its manufacturer, is not guaranteed or endorsed by the publisher.

## References

[B1] Ba L. Ning J. X. Wang D. L. Facelli E. Facelli J. M. Yang Y. N.. (2012). The relationship between the diversity of arbuscular mycorrhizal fungi and grazing in a meadow steppe. Plant Soil 352 (1-2), 143–156. doi: 10.1007/s11104-011-0985-6

[B2] Bardgett D. Cook (1999). Below-ground herbivory promotes soil nutrient transfer and root growth in grassland. Ecol. Lett. 2 (6), 357–360. doi: 10.1046/j.1461-0248.1999.00001.x

[B3] Bardgett R. D. Wardle D. A. Yeates G. W. (1998). Linking above-ground and below-ground interactions: how plant responses to foliar herbivory influence soil organisms. Soil Biol. Biochem. 30 (14), 1867–1878. doi: 10.1016/S0038-0717(98)00069-8

[B4] Belsky A. J. (1986). Does herbivory benefit plants? a review of the evidence. Am. Nat. 127 (6), 870–892. doi: 10.1086/284531

[B5] Cai D. W. Ge Q. S. Wang X. M. Liu B. L. Goudie A. S. Hu S. (2020). Contributions of ecological programs to vegetation restoration in arid and semiarid China. Environ. Res. Lett. 15 (11), 114046. doi: 10.1088/1748-9326/abbde9

[B6] Chen L. L. Wang K. X. Baoyin T. (2021). Effects of grazing and mowing on vertical distribution of soil nutrients and their stoichiometry (C: N: P) in a semi-arid grassland of north China. Catena 206, 105507. doi: 10.1016/j.catena.2021.105507

[B7] Clegg C. D. (2006). Impact of cattle grazing and inorganic fertiliser additions to managed grasslands on the microbial community composition of soils. Appl. Soil Ecol. 31 (1), 73–82. doi: 10.1016/j.apsoil.2005.04.003

[B8] Deng L. Sweeney S. Shangguan Z.-P. (2014). Grassland responses to grazing disturbance: plant diversity changes with grazing intensity in a desert steppe. Grass Forage Sci. 69 (3), 524–533. doi: 10.1111/gfs.12065

[B9] Duan M. Gao Q. Wan Y. Li Y. Guo Y. Luobu D.. (2010). Effect of grazing on community characteristics and species diversity of stipa purpurea alpine geassland in northern Tibet. Acta Ecologica Sin. 30 (14), 3892–3900.

[B10] Egan G. Zhou X. Wang D. M. Jia Z. J. Crawley M. Fornara D. A. (2018). Long-term effects of grazing, liming and nutrient fertilization on the nitrifying community of grassland soils. Soil Biol. Biochem. 118, 97–102. doi: 10.1016/j.soilbio.2017.12.005

[B11] Eldridge D. J. Delgado-Baquerizo M. Travers S. K. Val J. Oliver I. Hamonts K.. (2017). Competition drives the response of soil microbial diversity to increased grazing by vertebrate herbivores. Ecology 98 (7), 1922–1931. doi: 10.1002/ecy.1879 28459141

[B12] Epelde L. Lanzen A. Mijangos I. Sarrionandia E. Anza M. Garbisu C. (2017). Short-term effects of non-grazing on plants, soil biota and aboveground-belowground links in Atlantic mountain grasslands. Sci. Rep. 7 (1), 1–11. doi: 10.1038/s41598-017-15345-1 PMC5678074 29118337

[B13] Fichtner A. von Oheimb G. Haerdtle W. Wilken C. Gutknecht J. L. M. (2014). Effects of anthropogenic disturbances on soil microbial communities in oak forests persist for more than 100 years. Soil Biol. Biochem. 70, 79–87. doi: 10.1016/j.soilbio.2013.12.015

[B14] Gao Y. Han X. Wang S. (2004). The effects of grazing on grassland soils. Acta Ecologica Sin. 04), 790–797.

[B15] Gu W. (2013). Effects of seasonal delaying grazing on vegetation and soil under different grazing intensities. master's degree, xinjiang agricultural university.

[B16] He D. Bi J. Sha Y. Huang Z. (2009). Functional diversity of soil microbial community under different types of vegetation in the desert grassland. Agricultural Research in the Arid Areas (05), 149–155.

[B17] Hibbing M. E. Fuqua C. Parsek M. R. Peterson S. B. (2010). Bacterial competition: surviving and thriving in the microbial jungle. Nat. Rev. Microbiol. 8 (1), 15–25. doi: 10.1038/nrmicro2259 19946288 PMC2879262

[B18] Hou F. Yang Z. (2006). Effects of grazing of livestock on grassland. Acta Ecologica Sin. 01), 244–264.

[B19] Hu J. Hou X-Y Wang Z. Ding Y. Li X-L Li P.. (2015). Effects of mowing and grazing on soil nutrients and soil microbes in rhizosphere and bulk soil of stipa grandis in a typical steppe. Ying yong sheng tai xue bao = J. Appl. Ecol. 26 (11), 3482–3488. 26915206

[B20] Klumpp K. Fontaine S. Attard E. Le Roux X. Gleixner G. Soussana J.-F. (2009). Grazing triggers soil carbon loss by altering plant roots and their control on soil microbial community. J. Ecol. 97 (5), 876–885. doi: 10.1111/j.1365-2745.2009.01549.x

[B21] Li W. (1999). The evaluation of the research on the grazing optimization hypothesis. Grassland China 04), 62–67.

[B22] Li Y. Dong S. K. Gao Q. Z. Zhang Y. Liu S. L. Swift D.. (2019). Grazing promotes plant functional diversity in alpine meadows on the qinghai-Tibetan plateau. Rangeland J. 41 (1), 73–81. doi: 10.1071/rj18091

[B23] Liu Y Liu Z. Deng L. Wu G. (2016). ). species diversity and functional groups responses to different seasonal grazing in alpine grassland. Pratacultural Sci. 33 (07), 1403–1409.

[B24] McNaughton S. J. (1979). Grazing as an optimization process: Grass-ungulate relationships in the Serengeti. Am. Nat. 113 (5), 691–703. doi: 10.1086/283426

[B25] Milchunas D. G. Lauenroth W. K. (1993). Quantitative effects of grazing on vegetation and soils over a global range of environments. Ecol. Monogr. 63 (4), 327–366. doi: 10.2307/2937150

[B26] Mysterud A. Hessen D. O. Mobaek R. Martinsen V. Mulder J. Austrheim G. (2011). Plant quality, seasonality and sheep grazing in an alpine ecosystem. Basic Appl. Ecol. 12 (3), 195–206. doi: 10.1016/j.baae.2011.03.002

[B27] Nelson C. E. Carlson C. A. (2012). Tracking differential incorporation of dissolved organic carbon types among diverse lineages of Sargasso Sea bacterioplankton. Environ. Microbiol. 14 (6), 1500–1516. doi: 10.1111/j.1462-2920.2012.02738.x 22507662

[B28] Owen D. F. Wiegert R. G. (1976). Do consumers maximize plant fitness? Oikos 27 (3), 488–492. doi: 10.2307/3543467

[B29] Owen D. F. Wiegert R. G. (1981). Mutualism between grasses and grazers: An evolutionary hypothesis. Oikos 36 (3), 376–378. doi: 10.2307/3544637

[B30] Pulungan M. A. Suzuki S. Gavina M. K. A. Tubay J. M. Ito H. Nii M.. (2019). Grazing enhances species diversity in grassland communities. Sci. Rep. 9. doi: 10.1038/s41598-019-47635-1 PMC6671982 31371753

[B31] Qu T. B. Du W. C. Yuan X. Yang Z. M. Liu D. B. Wang D. L.. (2016). Impacts of grazing intensity and plant community composition on soil bacterial community diversity in a steppe grassland. PloS One 11 (7), e0159680. doi: 10.1371/journal.pone.0159680 27467221 PMC4965099

[B32] Song L. Y. Pan Y. Gong J. R. Li X. B. Liu M. Yang B.. (2020). Physiology of leymus chinensis under seasonal grazing: Implications for the development of sustainable grazing in a temperate grassland of inner Mongolia. J. Environ. Manage. 271. doi: 10.1016/j.jenvman.2020.110984 32579531

[B33] Steinauer K. Chatzinotas A. Eisenhauer N. (2016). Root exudate cocktails: the link between plant diversity and soil microorganisms? Ecol. Evol. 6 (20), 7387–7396. doi: 10.1002/ece3.2454 28725406 PMC5513276

[B34] Sternberg M. Golodets C. Gutman M. Perevolotsky A. Ungar E. D. Kigel J.. (2015). Testing the limits of resistance: a 19-year study of Mediterranean grassland response to grazing regimes. Global Change Biol. 21 (5), 1939–1950. doi: 10.1111/gcb.12866 25580928

[B35] Suo C. Fei X. Liu Y. Xiang S. Sun S. Functional group characteristics of plant community at different grazing intensities in alpine grassland of northwestern sichuan. Chin. J. Appl. Environ. Biol. 28 (06), 1–10. doi: 10.19675/j.cnki.1006-687x.2021.09044

[B36] Thukral A. (2017). A review on measurement of alpha diversity in biology. Agric. Res. J. 54, 1. doi: 10.5958/2395-146X.2017.00001.1

[B37] Van Syoc E. Albeke S. E. Scasta J. D. van Diepen L. T. A. (2022). Quantifying the immediate response of the soil microbial community to different grazing intensities on irrigated pastures. Agriculture Ecosyst. Environ. 326, 107805. doi: 10.1016/j.agee.2021.107805 PMC8782393 35068628

[B38] Wang X. Y. Li F. Y. Wang Y. N. Liu X. M. Cheng J. W. Zhang J. Z.. (2020). High ecosystem multifunctionality under moderate grazing is associated with high plant but low bacterial diversity in a semi-arid steppe grassland. Plant Soil 448 (1-2), 265–276. doi: 10.1007/s11104-020-04430-6

[B39] Wang L. Niu K. Yang Y. Zhou P. (2010). Patterns of above- and belowground biomass allocation in china’s grasslands: Evidence from individual-level observations. Sci. China Life Sci. 53 (7), 851–857. doi: 10.1007/s11427-010-4027-z 20697874

[B40] Wang S. Wang Y. (2001). Study on over-compensation growth of cleistogenes squarrosa population in inner mongolia steppe. J. Integr. Plant Biol. 04), 413–418.

[B41] Wardle D. A. Bardgett R. D. Klironomos J. N. Setala H. van der Putten W. H. Wall D. H. (2004). Ecological linkages between aboveground and belowground biota. Science 304 (5677), 1629–1633. doi: 10.1126/science.1094875 15192218

[B42] Xun W. B. Yan R. R. Ren Y. Jin D. Y. Xiong W. Zhang G. S.. (2018). Grazing-induced microbiome alterations drive soil organic carbon turnover and productivity in meadow steppe. Microbiome 6 (1), 1–13. doi: 10.1186/s40168-018-0544-y PMC6149009 30236158

[B43] Yang X. Shen Y. Badgery W. B. Guo Y. Zhang Y. (2018). Arbuscular mycorrhizal fungi alter plant community composition along a grazing gradient in inner Mongolia steppe. Basic Appl. Ecol. 32, 53–65. doi: 10.1016/j.baae.2018.07.002

[B44] Yang X. Zang J. Feng J. Shen Y. (2022). High grazing intensity suppress soil microorganisms in grasslands in China: A meta-analysis. Appl. Soil Ecol. 177, 104502. doi: 10.1016/j.apsoil.2022.104502

[B45] Zechmeister-Boltenstern S. Keiblinger K. M. Mooshammer M. Peñuelas J. Richter A. Sardans J.. (2015). The application of ecological stoichiometry to plant–microbial–soil organic matter transformations. Ecol. Monogr. 85 (2), 133–155. doi: 10.1890/14-0777.1

[B46] Zhang H. R. Fu G. (2021). Responses of plant, soil bacterial and fungal communities to grazing vary with pasture seasons and grassland types, northern Tibet. Land Degradation Dev. 32 (4), 1821–1832. doi: 10.1002/ldr.3835

[B47] Zhang C. Wang J. Liu G. Song Z. Fang L. (2019). Impact of soil leachate on microbial biomass and diversity affected by plant diversity. Plant Soil 439 (1), 505–523. doi: 10.1007/s11104-019-04032-x

[B48] Zhang Z. Zhou H. Zhao X. Yao B. Ma Z. Dong Q.. (2018). Relationship between biodiversity and ecosystem functioning in alpine meadows of the qinghai-Tibet plateau. Biodiversity Sci. 26 (02), 111–129. doi: 10.17520/biods.2017021

[B49] Zheng B. (2020). Effects of grazing seasons on the vegetation and soil of the typical steppe in inner mongolia: an study. master's degree, inner Mongolia university.

