# Supplementary material for: Effects of seasonal grazing on plant and soil microbial diversity of typical temperate grassland
Source: Front Plant Sci. 2022 Nov 3;13:1040377. doi: 10.3389/fpls.2022.1040377 (PMC9670318; doi:10.3389/fpls.2022.1040377)
Supplement: Supplementary file 1 [file DataSheet_1.docx]

# Supporting Information

# Supporting Information

## Method S1: *Diversity Index and its implications*

Shannon（*H'*）was determined by the following equation(Shannon et al.,1949)

$$H'=-\sum_{i=1}^{S} P_{i}\ln P_{i}$$

where *P_i_* represents the relative abundance of plant species *i* in one particular quadrat. *P_i_* was calculated as follows: *P_i_*=*n_i_/N*, where *n_i_* represents the number of individuals of plant species *i* and *N* represents the total number of individuals of all plant species in one particular quadrat, respectively. The index reflects community species diversity based on the number of species: an increase in biological species in a community represents an increase in the complexity of the community, i.e. the greater the *H*′value, the greater the amount of information contained in the community.

Simpson（*D*）was determined by the following equation(Simpson et al.,1949)

$$D=\sum_{i=1}^{S} (\frac{n_{i}}{N})^{2}$$

where n_i_ represents the number of individuals of plant species *i* and *N* represents the total number of individuals of all plant species in one particular quadrat, respectively. It is often used in ecology to quantitatively describe the biodiversity of an area.

Pielou（*E_H_*）was determined by the following equation(Pielou,1966)

$$E_{H}=\frac{H'}{\ln S}$$

where *S* represents the number of plant species in one particular quadrat. This index is the use of species singularity (*H'*) to estimate the uniformity of species distribution in this community.

Margalef（*F*）was determined by the following equation(Margalef,1951)

$$F=\frac{S-1}{\ln N}$$

where *S* represents the number of plant species and N represents the total number of individuals of all plant species in one particular quadrat, respectively. This index reflects the species richness of the community: it refers to the number of species in a community or environment, and also indicates the index of the degree of species richness in the biome (or sample).

Chao（*S_chao1_*）was determined by the following equation(Chao,1984)

$$S_{chao1}=S_{obs}+\frac{n_{1}(n_{1}-1)}{2(n_{2}+1)}$$

where *S_chao1_* represents the estimated number of OUTs, S_obs_ represents the number of OUT observed, *n_1_* represents the number of OUT with only one sequence, and *n_2_* represents the number of OUT with only two sequences. The index was used to assess the number of OTU in a sample, and the larger the Chao index, the greater the number of OTUs, indicating that the sample had a larger number of species.

Shannoneven was a Shannon index-based measure of evenness，this index is used to indicate the uniformity of individual distributions within a species .Coverage refers to the coverage of each sample (clone) library, the higher the value, the higher the probability of the sequence being measured in the sample, and the lower the probability of not being measured. This index reflects whether the results of this sequencing represent the true condition of the microorganisms in the sample.

**References：**

Chao, A. (1984). Non-parametric estimation of the number of classes in a population. Scandinavian Journal of Statistics, 11:265-270

Margalef R. (1951). Diversidad de especies en las comunidades naturales (Vol. 6, pp. 59‒72). Barcelona: Publicaciones del Instituto de Biologia Aplicada.

Pielou E.C. (1966). The measurement of diversity in different types of biological collections. Journal of Theoretical Biology 13: 131‒144.

Shannon, C.E., and Weaver, W. (1949). The Mathematical Theory of Communication. University of Illinois Press, Urbana, Illinois, pp. 1‒117.

Simpson, E.H. (1949). Measurement of diversity. Nature 163, 688.
